# Completion of maternity continuum of care among women in the post-partum period: Magnitude and associated factors in the northwest, Ethiopia

**DOI:** 10.1371/journal.pone.0237980

**Published:** 2020-08-27

**Authors:** Melaku Hunie Asratie, Achenef Asmamaw Muche, Alehegn Bishaw Geremew

**Affiliations:** 1 Department of Women’s and Family Health, School of Midwifery, College of Medicine and Health Sciences, University of Gondar, Gondar, Ethiopia; 2 Department of Epidemiology and Biostatistics, Institute of Public Health, College of Medicine and Health Sciences, University of Gondar, Gondar, Ethiopia; 3 Department of Reproductive Health, Institute of Public Health, College of Medicine and Health Sciences, University of Gondar, Gondar, Ethiopia; University of Cape Coast, GHANA

## Abstract

**Background:**

Maternity continuum of care is a model of integrated components of maternal health service from pregnancy to the post-partum period to improve maternal, neonatal and child health. In Ethiopia, the magnitude of antenatal care, skilled delivery, and post-natal care have shown improvement. However, there is limited evidence of the woman who attends continuing from antenatal care to post-partum care.

**Objective:**

To assess completion of maternity continuum of care and its associated factors among women, in Motta town and Hulet Eji Enese district, Northwest Ethiopia.

**Methods:**

A community based cross-sectional study with a stratified cluster sampling technique was conducted among 819 women 6week-6month post-partum period in Motta town and Hulet Eji Enese district. The data were collected from March 12, 2019 to April1, 2019 by face to face interviews, using a pretested structured questionnaire. Binary logistic regression (bi-variable and multivariable) model was done. Adjusted odds ratio with respect to 95% confidence interval was employed in the strength and direction of the association between covariates and outcome variable. Besides, a P value<0.05 was used to declare statistical significance.

**Results:**

A total of 819 women with100% response rate participated and Completion of maternity continuum of care was found to be 47% (43.2%-50.2%) in the study. Educational attainment of Secondary school and above (adjusted odds ratio(AOR) = 3.5; 1.9–6.3), urban residence (AOR = 4.6; 95%CI 2.5–8.5), women reach to a health facility within 30minute(AOR = 2.1; 95%CI 1.2–3.7), a woman was the primary decision maker for attending maternity continuum of care(AOR = 3.5;95%CI 1.9–6.3), index pregnancy-related complication(AOR = 2.4;95%CI 1.1–5.3), starting antenatal care within second trimester (AOR = 3.4;95%CI 2.1–5.6) and antenatal care visit 3–4 times(AOR = 2.1;95%CI 1.2–3.8) were statistically significant with completion of maternity continuum of care.

**Conclusions:**

The completion of maternity continuum of care is low in the study area. Improving the educational status of women, engaging the rural community, physical accessibility of health facility, woman empowerment for decision making, emphasis on giving care for pregnancy-related complication, and early gestational age antenatal care at least 3 or more visits suggested to increase completion of maternity continuum of care.

## Background

Continuum of care is a model for continuously giving health care services. its concept 1^st^ comes to light in 1970 with relation to integrating research and practice to providing smooth collaboration between care-providing organizations and professions to create a continuum of care for frail older people [1, 2]. Over the past decade, the World Health Organization (WHO) and other organizations had advocated continuum of care throughout the life-cycle, including adolescence, pregnancy, childbirth, and childhood related to time dimension to improve maternal, newborn, and child health (MNCH) [3–5]. Furthermore continuum of care had narrowed to time dimension as maternity continuum of care [6].

Maternity continuum of care is the continuation of care starting from pregnancy to the postpartum period. Among countries, including Ethiopia, there were greater priorities for maternal, neonatal, and child health since 2000–2015, as countries should not wait to start action without highly developed technology simply by a maternity continuum of care. For this safe motherhood and child survival programs had directed to cut maternal, neonatal, and child mortality through coördinated maternity care from pregnancy through antenatal care (ANC), during delivery through skilled birth attendant(SBA) and post-partum care(PPC) by health care practitioner [3, 7, 8]. This effort had shown an improvement especially on maternal and child health, but the completion of maternity continuum of care can solve the limited reduction in neonatal mortality [9].

The concept of completion of the maternity continuum of care has been worked to improve the status of maternal, neonatal, and child health in the field of global health [10]. Worldwide it is obvious that under five years of age children’s mortality had improved dramatically yet every day in 2016,15000 children died before celebrating their fifth birthday and 40% of them within 28 days [11]. In Ethiopia, the expansion in coverage of maternal and child health care services at the national level: Antenatal care (27%-62%), skilled birth attendant (5%-28%), and postnatal care (17%) had sown a great contribution for the decline of maternal mortality to 412 maternal deaths per 100,000 live births, and under-five mortality to 68 per 1000 live birth. But in the case of neonatal mortality still, no congenial improvement which shows 49 to 29 mortality per 1000 live birth [12].

Currently, Ethiopia is on the due date of finalizing health sector transformation plan which stands reproductive, maternal, neonatal, child and adolescent health (RMNCAH) as a top priority and compassionate, respectful and caring health professional with completion of maternity continuum of care as 3rd integrated transformational agenda [13]. Besides ending preventable maternal and child death through safe, more effective, accessible, equitable care for every woman by 2030 is the sustainable development goal of Ethiopia [9].

Completion of the maternity continuum of care can make up the sustainable development of goals especially in reducing neonatal morbidity and mortality [14]. There is an evidence-based, cost-effective intervention which shows universal (99%) coverage of completion of maternity continuum of care could avert an estimated 41–72% of neonatal death worldwide [15]. Another finding from systematic review and meta-analysis of 19 randomized and quasi-randomized controlled trials in low-income countries has revealed that completion of maternity continuum of care halts neonatal and perinatal mortality [16]. Despite, the plan and advantages of completion of maternity continuum of care there is still limited evidence on the progress of women full fill components of maternity continuum of care starting from antenatal care during pregnancy state, labor and delivery attended with a skilled health care provider and finally end with post-partum care within 6 weeks of post-partum period in the complete way. Moreover, the factors associated with completion of maternity continuum of care had not been studied in the previous findings. Therefore, this study aims to assess completion of maternity continuum of care and identifying factors associated with completion of maternity continuum of care.

## Methods

### Study design, area and period

A community-based cross-sectional-study was conducted from March 12/2019 to April 1/2019. The study was conducted in Motta town and Hulet Eji Enese district. Currently, there are 6 kebeles in Motta town and 30 kebeles in the Hulet Eji Enese district. Motta town has 1hospital, 1health center, 5 private clinics, and 4health posts whereas Hulet Eji Enese district has 8 health centers, 30 health posts, and 3 private clinics. Based on the data reported from Motta town health office the total population was around 31,483 of those 15,619 were male and 15,864 females. Whereas Hulet Eji Enese district 244,155 of those 121,078 were men and 123,077females. From the report of Motta town health office, there were 500 Pregnant women with the first visit of antenatal care, 260 women delivered at the health facility and 520 women had post-partum care (both health facility delivery and home delivery). On the other hand, two month report of Hulet Eji Enese district health office was 2288 pregnant women with first visit, 570Women delivered at the health facility and 480 women with post-partum care (both health facility delivery and home delivery).

### Sample size determination and sampling technique

The sample size was calculated using single population proportion formula by assuming that the estimated magnitude of completion of maternity continuum of care among women 6week-6month post-partum period and who had at least one antenatal care visit was 60% [17]. With a 5% margin of error and by assuming 2 design effects, and a 10% non-response rate the final required minimum sample size was estimated to be 812. From Motta town there are a total of 6 kebeles and 3 kebeles were taken by simple random sampling technique and from Hulet Eji Enese district 7 kebeles were selected from a total of 30 kebeles by simple random sampling technique, 10 cluster kebeles were included in the study from both Motta town and Hulet Eji Enese district. Then from each selected 10 cluster kebeles, all women within 6week-6month post-partum periods and who had at least one antenatal care had taken as study participants. Finally, we attain 819 study participants.

### Operational definition

The outcome variable of this study was completion of maternity continuum of care. Completion of maternity continuum of care was defined as whether a post-partum period woman having one or more ANC visits at the health facility during pregnancy, childbirth aided by SBA(doctor, nurse, and midwife, health officer, and health extension worker), and having one or more PNC for the mothers within 6 weeks after viable childbirth based on self-reports [18, 19].

### Data collection instrument and procedures

The data collection tool was a structured questionnaire adapted from the literatures. The questionnaire was first prepared in English and translated to Amharic (local language) then back to English to maintain consistency of the tool. Finally, the questionnaires were prepared in a local language, Amharic, to make it simple and understandable.

It has four categories. Socio-demographic characteristics, health care service-related factors, obstetrical related factors, and maternal health care service-related factors. The questionnaire was pretested in Bahirdar with 41(5%) participants to check the response, language clarity, and appropriateness. At the end of the pretest arrangements of questions were undertaken.

Three female BSc midwives for data collection and two male BSc midwives for supervision were assigned. One day training was provided for those data collectors and supervisors about the purpose of the study and techniques of data collection. The trained data collectors were supervised at the time of data collection and the questionnaire was checked for completeness on a daily basis.

### Data processing and analysis

The data were checked for completeness, coded, and then entered into EPI -info version 7.2 and transferred to the Statistical Package of Social Science (SPSS) version 22 for analysis. Descriptive statistics were expressed in numerical value, mean, standard deviation, median, interquartile range, and percentage. The data were presented using text, tables, and graphs. A binary logistic regression (bivariable and multivariable) was fitted to identify factors associated with completion of maternity continuum of care. Multivariable model was tested for its goodness of fit with the Hosmer-Lemeshow test (P value = 0.25) and the value was not significant.

All Variable from a bivariate analysis with a p value less than or equal to 0.2 was entered in multivariable analysis and backward likelihood ratio was used. Adjusted odds ratio with a 95% confidence interval was used to investigate the strength and directions of the association between covariates and completion of maternity continuum of care. Variables with P value ≤0.05 from the multivariable analysis were considered as significantly associated with completion of maternity continuum of care.

Ethical clearance was obtained from the Institutional Review Board (IRB) of the University of Gondar, the Institute of public health. A formal letter of approval was taken from Motta town administrative health office and Hulet Eji Enese district administrative health office. The purpose, risk, and benefits of the study was explained in detail. We told as participation was on a voluntary basis, and they can withdraw at any time if there is any inconvenience at the time of the interview, and verbal informed consent was obtained from every study participant before data collection. For participants age <18 verbal informed consent was taken from their parents and assent obtained from the minor/participant. And it was approved by the ethical review committee of the institute of public heath on behalf of IRB of University of Gondar.

## Results

### Socio-demographic characteristics of the study participants

A total of 819 women with a response rate of 100% participated in the study. Their mean age was 31 years± (SD 7.27 years) with 683 (83.4%) of them were Orthodox and 657 (80.2%) were married. All study participants were Amhara in Ethnicity with 634 (77.4%) of them from rural residences and 570 (69.6%) had no formal education. More than half 444 (54.2%) of the respondents were farmers by occupation. Two-third 517 (63.1%) of women partners had no formal education. Their median monthly income was 2400 EB with (IQ R of 1900-3286EB) Table 1.

**Table 1:** Socio-demographic characteristics of women and partners in Motta town and Hulet Eji Enese district, northwest Ethiopia; 2019.

Characteristics	Frequency	Percentage
**Age of woman in year**		
15–19	176	21.5
20–34	335	40.9
35–49	308	37.6
**Religion**		
Orthodox	683	83.4
Muslim	111	13.6
Protestant	13	1.6
Catholic	12	1.4
**Marital status**		
Married	657	80.2
Cohabiting	77	9.4
Separated	38	4.6
Divorced	11	1.4
Widowed	12	1.5
Single	24	2.9
**Educational status of woman**		
Has no formal education	570	69.6
Grade 1–8	69	8.4
Grade 9–12	88	10.7
College and above	92	11.3
**Residency**		
Rural	634	77.4
Urban	185	22.6
**Occupational status of women**		
Farmer	444	54.2
Housewife	155	18.9
Private employee	45	5.5
Government employee	50	6.1
Student	93	11.4
Others	32	3.9

Other = merchant, daily laborer

### Health care service-related characteristics of participants

Among 819 respondents 373(45.5%) of them were the primary decision maker for attending maternity continuum of care. Around 567 (69.2%) of them can get an ambulance for maternal health-related service and 425 (51.9%) of them need less than or equal to 30 minutes to reach the nearest health facility seeking health care.

### Obstetrical related factors for respondents

Among 819 respondents 564 (68.8%) of them were gravid 2–3 and 171(20.9%) of them had poor obstetric history through their reproductive age. The major type was Intra Uterine Fetal Death (IUFD) 83 (48.5%). Tiny fraction 83(10.1%) of them had developed obstetrical complications during their pregnancy period for their last baby, pregnancy-induced hypertension accounted for 59 (71.1%) of all complications.

### Maternal health services-related factors

Among 819 respondents 516 (63%) of them attended their 1^st^ ANC visit within the second trimester, 235 (28.7%) of them had ANC follow up 3. From all 819 extended post-partum period, women within 6months and those who had at least one ANC follow up 536 (65.4%) of them delivered at the health facility. For those who delivered in the Health facility, 373 (46%) had a postpartum check. Among 819 respondents 283 (34.6%) of them delivered at home and 12 (4.3%) of them attended by health care providers of those 10 (83.3%) of them had received post-partum care Table 2.

**Table 2:** Maternal health services-related factors among women in Motta Town and Hulet Eji Enese district, Northwest, Ethiopia; 2019.

Variables	Frequency	Percentage
**Gestational age during 1**^**st**^ **antenatal care**		
Within second trimester	516	63
Above second trimester	303	37
**Number of antenatal care visit**		
One time only	177	21.6
Two times	206	25.2
Three times	235	28.7
Four times	201	24.5
**Site for delivery**		
Health facility	536	65.4
Home	283	34.6
**Post-partum care from health facility delivery n = 536**		
Yes	373	45.5
No	163	19.9
**Birth attendant from home delivery**		
Health care professional	12	4.3
Traditional birth attendant	66	23.3
Herself	156	55.1
Others	49	17.3
**Post-partum care after skilled delivery at home n = 12**	10	83.3

Other** = mother, grandmother, sister

### Magnitude of completion of maternity continuum of care

**Fig 1:**
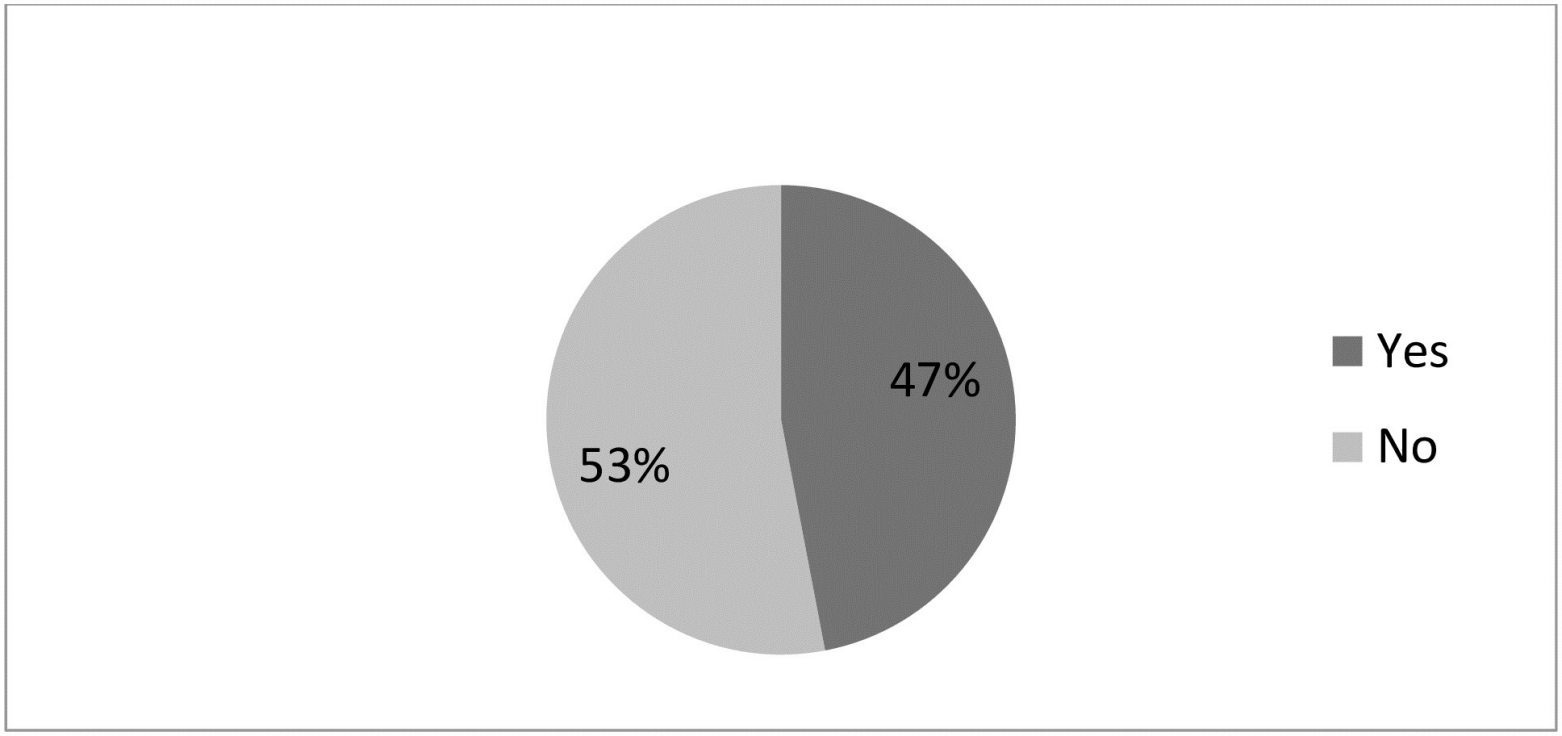
The magnitude of completion of maternity continuum of care among women who had antenatal care in Motta town and Hulet Eji Enese district northwest Ethiopia.

The magnitude of completion of maternity continuum of care was found to be 47% (95% CI 43.2%-50.2%) Fig 1. Of this 373(46%) of them complete maternity continuum of care with health facility delivery, whereas 10 (1%) respondents complete maternity continuum of care with home delivery.

### Factors associated with completion of maternity continuum of care

On bivariate analysis four socio-demographic variables educational status of both partners, occupational status of the woman, residency, and other seven variables were significantly associated with completion of maternity continuum of care. In multivariable analysis, seven variables educational status of the woman, residency, distance to the health facility, decision-maker for attending maternity continuum of care, index pregnancy-related complications, gestational age for starting ANC and number of antenatal cares, were continued to be significant with completion of maternity continuum of care.

Respondents with educational status of secondary and above were 4 times more likely to complete maternity continuum of care (AOR = 3.5; 95% CI 1.9–6.3) as compared to those have no formal education, women in the urban residence were 5 times more likely to complete maternity continuum of care (AOR = 4.6; 95% CI 2.5–8.5) as compared to those rural, women who respond can reach to a health facility within 30 minute are 2 times more likely to complete maternity continuum of care (AOR = 2.1;95% CI 1.2–3.7) as compared to women said distance to health facility takes more than 30 minutes.

Woman was the primary decision maker for attending maternity continuum of care were 4 times more likely to complete maternity continuum of care (AOR = 3.5;95% CI 1.9–6.3) as compared to those the woman was not the primary decision maker, women who develop pregnancy-related complication during indexpregnancy were 2 times more likely to complete maternity continuum of care (AOR = 2.4;95% CI 1.1–5.3) as compared to those normal state, respondents antenatal care visit start within the second trimester were 3 times more likely to complete maternity continuum of care (AOR = 3.4; 95% CI 2.1–5.6) as compared to those start their ANC above second trimester and respondents with antenatal care visit 3–4 were 2 times more likely to complete maternity continuum of care (AOR = 2.1; 95% CI1.2–3.8) as compared to those with ANC visit 1–2 Table 3.

**Table 3:** Bivariable and multivariable logistic regression analysis of factors associated with completion of maternity continuum of care among women in Motta town and Hulet Eji Enese district, Northwest; Ethiopia; 2019.

Variables	Completion of maternity continuum of care		COR 95%CI	AOR 95%CI
	Yes	No		
**Educational status of woman**				
No formal education	211	359	1	1
≤grade 8	37	32	1.9(1.2–3.3)***	2.1(0.6–6.8)***
≥grade 9	135	45	5.1(3.5–7.4)***	3.5(1.9–6.3)***
**Occupation of woman**				
Farmer	162	282	1	NA
Housewife	77	78	1.7(1.2–2.5)**	
Private employee	33	12	4.8(2.4–9.5)***	
Government employee	33	17	3.4(1.8–6.3)***	
Student	57	36	2.7(1.5–7.1)***	
Others	21	11	3.3(1.7–4.4)**	
**Residency**				
Rural	244	390	1	1
Urban	139	46	4.8(3.3–6.9)***	4.6(2.5–8.5)***
**Husband/partner education**				
No formal education	192	325	1	NA
≤grade 8	14	21	1.1(0.6–2.2)**	
≥grade 9	177	90	3.3(2.4–4.5)***	
**Decision maker for attending maternity continuum of care**				
Woman was primary decision maker	316	86	19(13–27)***	3.5(1.9–6.5)***
The woman was not the primary decision maker	67	350	1	1
**Distance interims of time it takes**				
≤30minute	317	108	15(10–20)***	2.1(1.2–3.7)**
≥30minute	66	328	1	1
**Ambulance for obstetrical emergency**				
Yes	341	226	7.5(5–10)***	NA
No	42	210	1	
**Number of pregnancy**				
1	58	44	1	NA
2–3	266	298	0.6(0.4–1)**	
4+	59	94	0.5(0.3–0.8)***	
**Pregnancy related complication**				
Yes	57	26	2.7(1.7–4.5)***	2.4(1.1–5.3)**
No	326	410	1	**1**
**Gestational age during 1**^**st**^ **antenatal care**				
Within second trimester	335	181	10(7–14)***	3.4(2.1–5.6)***
Above second trimester	48	255	1	**1**
**Number of antenatal care**				
≤2	52	331	1	**1**
3–4	331	105	20(13–28)***	2.1(1.2–3.8)**

COR: crude odds ratio AOR: adjusted odds ratio, CI: confidence interval NA = not have association with outcome variable 1: reference category, other* = merchant, daily laborer other** = student, daily laborer.

* = 0.05<p<0.2,

** = 0.001<p<0.05,

*** = p<0.001

## Discussion

Assessing the health status of the women starting from pregnancy state, labor and delivery and post-partum period through a model of maternity continuum of care is considered to be the most effective approach to achieve sustainable development goal of Ethiopia which is entitled with ending preventable cause of maternal, neonatal and child death by 2030.

The study finds that the overall completion of maternity continuum of care is 47%(43.2%-50.2%). This is lower than that reported in the previous study in Cambodia(60%) [19]. This could be due to the strength of program efforts of the government of Cambodia in collaboration with multiple international development partners to improve maternal health care [20].

On the other Hand Completion of maternity continuum of care in our study is in line with the findings from Nepal (45.7%) [21] and much higher as compared to study in Pakistan(27%) [22], sub-Saharan Africa(13.6%) [23]. This significantly higher completion of maternity continuum of care in our study might be attributed to the inclusion of women who received at least one antenatal care, labor, and delivery with skilled birth attendant and post-partum care within 6 weeks of the post-partum period. Which means a woman with only one ANC visit can be considered as complete maternity continuum of care as she has attended by a skilled provider during labor and end with post-partum care. Another possible explanation our finding is based on post-partum check within 6 weeks of the post-partum period this leads to the inflated magnitude as compared to studies with operational definition post-partum care within 48 hours. Similarly finding from our study is significantly higher than the study conducted in Nigeria (11.7%) [24], Ghana(8%) [25], and Tanzania (10%) [18]. The possible explanation could be the difference in sample size for comparison of the outcome variable in Nigeria is larger since it was DHS data, Ghana women’s highly influenced by cultural belief which means a woman perceives the cause of a health problem as spiritual rather than physical [25, 26]. For this delivery by traditional birth, attendant is highly practiced by precluding a laboring mother from joining to the health facility and have an access skilled birth attendant. On the other hand, even a woman who delivered to the health facility may not need to return for post-partum care within 6 weeks after once they did not get immediately at the health facility. Finally, the possible explanation for higher finding as compared to evidence in Tanzania could be the nature of inquiry was focused on the contact points across the continuum of care, since pre-discharge care after childbirth was considered as part of the ‘delivery care’ contact (not as a PPC) whereas in our study postpartum care starting 2hr after delivery to 6-week post-partum period.

Regarding the factors associated with completion of maternity continuum of care in this study, educational status of the woman, residency, distance to the health facility, decision -maker for attending maternity continuum of care, complication during the index pregnancy, gestational age starting antenatal care, number of antenatal cares, health care provider support during last antenatal care are variables which determine completion of maternity continuum of care.

Respondents secondary and above educational status are nearly four time complete maternity continuum of care as compared to those who have no formal education. This finding is supported by studies conducted in South Asia and sub-Saharan Africa as women with educational status of secondary and above six times complete maternity continuum of care as compared to those who have no formal education [10]. Similarly, there are different studies which show increasing educational status of the woman positively associated with completion of maternity continuum of care [6, 24, 25, 27–32]. The possible explanation could be women who have no education cannot understand the formal communication with the health care providers at the time of ANC either for birth preparedness or complication readiness. On the other hand, educated women are familiar with the meaning and importance of maternal health care services that may have a good chance to approach the written information and may have more cultural perspectives to cooperate with healthcare providers.

Women whose residence is urban five times complete maternity continuum of care as compared to who reside in rural. This finding is supported by evidence from different studies [10, 19, 25, 33]. The possible explanation might be women in the urban community are much more likely to be educated [9]. In turn educational status of the women is significantly associated with completion of maternity continuum of care in our study. The other possible explanation might be women in urban have better access to a large number of health facility and health care providers as well this may lead to increase in the chance of availing throughout the continuum of care.

Women respond reach health facility within a 30minute for attending maternity continuum of care are two times complete maternity continuum of care as compared to those respond reach to heath facility >30minute for attending maternity continuum of care. This finding is supported by evidence from a study done Jima zone respondents reside with <30minute to reach health facility two times Complete maternity continuum of care as compared to respondents reside with >60minute takes to reach health facilities [33, 34]. It might be due to delays in accessing health care services since delayed to reach health facility is the second model among the 3 delayed models for maternal mortality, especially due to lack of skilled delivery which is one component of maternity continuum of care.

Respondents who can decide by them self’s for attending maternity continuum of care is four times complete maternity continuum of care as compared to respondents who cannot decide by them self’s for attending maternity continuum of care. This finding is supported by a study done in Cambodia [27]. The possible explanation could be women who cannot decide for maternal health services mostly exposed to upland and unwanted pregnancy. This type of pregnancy mostly associated with less to attend the full elements of maternity continuum of care [25].

Respondents who developed pregnancy-related complication during index pregnancy 2.4 times complete maternity continuum of care as compared to those in normal state. This evidence is supported by a study done in Nepal which shows women with pregnancy-related complication 1.4 times complete maternity continuum of care as compared to women normal state [35]. This might be due to increased contact rate to health care provider secondary to the appointment for follow up care or medical treatment.

Respondents with Gestational age ≤24 for starting antenatal care were3.4 times complete maternity continuum of care as compared to respondents antenatal care visit start ≥25weeks. This finding is supported by a study done in Cambodia, Tanzania [18, 27]. The possible explanation for this could be respondents who start antenatal care in the early gestational age has higher chance to contact with the health care provider and have access for adequate information like birth preparedness and complication readiness for the subsequent components of maternity continuum of care like skilled delivery and post-partum care.

Respondents with the number of antenatal care 3–4 are two times to complete maternity continuum of care as compared to the respondent with antenatal care 1–2. This finding is supported by different studies [10, 18, 36–38]. The possible explanation could be women with a greater number of antenatal care visit have greater contact with health care provider this can be important to gate the full services of antenatal care including birth preparedness and complication readiness then a woman who prepared for birth have a higher chance to avail on time to the health institution and delivered by skilled birth attendant and having immediate post-partum care as well. Antenatal care is noteworthy predictor of seeking assistance during delivery by skilled personnel and having post-natal care [29].

## Strength and weakness of the study

The study participants were extended post-partum period women in that they were asked to recall their pregnancy experience, recall bias was possible and we had tried to minimize by limiting the source population within 6 week-6month post- partum period and we believe it is the best method to collect information on MNCH service based from pregnancy to postnatal care rather from the recall response of the respondent. Second, since the data were collected through the interviewer administered questionnaire, social desirability bias was possible and we had tried to address by making the data collectors from different site.

## Conclusions

This study demonstrated that completion of maternity continuum of care was found to be low. Educational status of the woman secondary and above, urban residence, reach to a health facility within 30 minutes, women decision-maker for attending maternity continuum of care, index pregnancy-related complication, starting antenatal care within second trimester, number of antenatal cares 3–4 were positively associated with completion of maternity continuum of care. Thus, we recommend Policymakers and implementers to engage improving secondary and above educational status of women, especially those reside in rural community and increasing their decision making power for attending maternity continuum of care. Great attention should be given for early initiation of antenatal care with increasing the number of visits.

## Supporting information

S1 Annex (DOCX)

S2 Annex (DOCX)

S1 Data (SAV)

## Abbreviations

ANC: Antenatal Care
AOR: Adjusted Odds Ratio
CI: Confidence Interval
COR: Crude Odd Ratio
EDHS: Ethiopia Demographic and Health Survey
MNCH: Maternal, Neonatal and Child Health
PPC: Post-Partum Care
SBA: Skilled Birth Attendance
SDG: Sustainable Development Goal
SPSS: Statistical Package for Social Science
WHO: World Health Organization
