# Supplementary material for: Completion of maternity continuum of care among women in the post-partum period: Magnitude and associated factors in the northwest, Ethiopia
Source: PLoS One. 2020 Aug 27;15(8):e0237980. doi: 10.1371/journal.pone.0237980 (PMC7451525; doi:10.1371/journal.pone.0237980)
Supplement: S1 Annex — (DOCX) [file pone.0237980.s001.docx]

**Annex II:-Questionnaire English Version**

Instruction: please encircle the number listed before the option to indicate your response and fill the blank for without option.

| Part I: socio- demographic factors | | | | |
| --- | --- | --- | --- | --- |
| S/n | | Questions | Answers/choices | Skip |
| 101 | | What is your age? | ___________complete years |  |
| 102 | | What is your religion? | 1. Orthodox  2. Muslim  3. Protestant  4. Catholic  5. Others (Specify) |  |
| 103 | | What is your marital status? | 1. Married 2. Cohabiting 3. Separated 4. Divorced 5. Widowed 6. Single |  |
| 104 | | What is your educational status? | 1. No formal education  2. Primary education (1-8)  3. Secondary education (9-12)  4. College and above |  |
| 105 | | Ethnicity | 1. Amhara  2. Tiger  3. Oromo  4. Others specify |  |
| 106 | | What is your occupation? | 1. Farmer 2. Housewife 3. Private employee 4. Government employee 5. Merchant 6. Student 7. Day laborer |  |
|  | |  | 8. Others specify______ |  |
| 107 | | Where is your residency? | 1. 1. Urban 2. 2. Rural |  |
| 108 | | What is your partner Educational status? | 1. No formal Education  2. Primary education (1-8)  3. Secondary education (9-12)  4. College and above |  |
| 109 | | What is your partner's occupation? | 1. Farmer 2. Private employee 3. Government employee 4. Merchant 5. Student 6. Day laborer 7. Other, specify--- |  |
| 200 | Average family monthly income | | ----------ETB complete |  |
| **Part ll: health care service-related factor** | | | | |
| 201 | Who is the primary decision maker from your family members for attending maternity continuum of care? | | 1. Woman herself 2. Husband 3. Others |  |
| 202 | How far the health institution from your home in terms of time you spent to reach there | | ---- |  |
| 203 | Is ambulance well arranged for emergent pregnancy related condition? | | 1. Yes 2. No |  |

| 300 | How many times have you had pregnant? |  |  |
| --- | --- | --- | --- |
| 301 | Have you poor obstetric history before index baby? | 1. yes  2. no | If no go to Q 303 |
| 302 | If yes in Q no 301 which problem?  More than one answer possible | 1. Abortion 2. Intra uterine fetal death 3. Stillbirth 4. Early neonatal loss |  |
| 303 | Have you ever faced any of maternal morbidity during index pregnancy period? | 1. Yes 2. No | If no, skip to Q 401 |
| 304 | If yes, which common morbidity?  More than one answer possible | 1. Antepartum hemorrhage 2. Pregnancy induced hypertension 3. Other, specify… |  |
| **Part Iv: maternal health services related** | | | |

**Part III: Obstetrics/ individual reproductive related question**

| 401 | At what time did you identify your pregnancy status for your index pregnancy? | 1. …. Week |  |
| --- | --- | --- | --- |
| 402 | At what time have you started first visit? | ----Week |  |
| 403 | How many times did you receive antenatal care? | -----Number |  |
| 404 | Where did you give birth your last baby? | 1. Institution 2. Home 3. Other, specify | If you deliver other than a health institution go to Q 407 |
| 405 | Did anyone check on your health while you were still in the facility or after you have left the health facility? | 1. Yes 2. No |  |
| 406 | If the answer is yes from Q 405 when did you receive your post-natal check? More than one choose is possible. | 1. 24 HR  2. 2-3 days  3. 6-7 day  4. At 6 weeks |  |
| 407 | By whom did you assist during delivery of your index baby? | 1. Doctor.  2. Nurse  3. Midwife  4. Health extension worker  5. Traditional birth attendant |  |
| 408 | Did you receive a postnatal check on your last baby’s? | 1. Yes 2. No |  |
| 409 | If the answer is yes from Q no 408 When did you receive your post-natal check? More than one choose is possible. | 1. 24 HR  2. 2-3 days  3. 6-7 days  4. At 6 weeks |  |
| 500 | Where did the postnatal care take place? | 1. Health post 2. Health center   3. Public hospital  4. Private health institution |  |
|  | The woman had at least one ANC, SBA and have at least one PNC | 1. Complete 2. Not complete |  |
