# Supplementary material for: Completion of maternity continuum of care among women in the post-partum period: Magnitude and associated factors in the northwest, Ethiopia
Source: PLoS One. 2020 Aug 27;15(8):e0237980. doi: 10.1371/journal.pone.0237980 (PMC7451525; doi:10.1371/journal.pone.0237980)
Supplement: S2 Annex — (DOCX) [file pone.0237980.s002.docx]

Annex IV የአማርኛ መጠይቅ

መመሪያ፡ ከተዘረዘሩት ምርጫዎች መልስ የያዘዉን ቁጥር በማክበብ እና ምርጫ ለሌላቸዉ ጥያቄዎች ክፍት ቦታዉን በመሙላት ይመልሱ።

**ክፍል1፡-ማህበራዊ ነክ መረጃዎችን በተመለከተ**

| **ተራቁጥር** | | ጥያቄወች | | መልስ | ይለፉ | |  |
| --- | --- | --- | --- | --- | --- | --- | --- |
| 101 | | እድሜዎት ስንት ነዉ? | | ----አመት |  | |  |
| 102 | | ሐይማኖትዎት ምንድን ነዉ? | | 1. ኦርቶዶክስ  2. ሙስሊም  3. ፕሮቴስታንት  4. ካቶሊክ  5. ሌላ ይግለፁ |  | |  |
| 103 | | የጋብቻ ሁኔታ | | 1. ያገባች 2. ሳይጋቡ አብረዉ የሚኖሩ 3. ተለያይተዉ የሚኖሩ 4. የፈታች 5. የሞተባት 6. ያላገባች |  | |  |
| 104 | | የትምህርት ደረጃዎት? | | 1. መደበኛ ትምህርት የሌላቸዉ  2. አንደኛ ደረጃ(1-8)  3. ሁለተኛደረጃ(9-12)  4. ኮሌጅ እና ከዚያበላይ |  | |  |
| 105 | | ብሄርዎ ምንድን ነዉ ? | | 1. አማራ  2. ትግሬ  3. ኦሮሞ  4. ሌላ ካለ ይገለፅ-- |  | |  |
| 106 | | የመኖሪያ ቦታ | | 1. ገጠር 2. ከተማ |  | |  |
| \|  \| \| --- \| \| 107 \| የስራ ሁኔታዎት ምንድን ነዉ? \| 1. አርሶ አደር 2. የቤት እመቤት 3. የግል ተቀጣሪ 4. የመንግስት ተቀጣሪ 5. ነጋዴ 6. ተማሪ 7. የቀን ሰራተኛ 8. ሌላ ይግለፁ--- \|  \| \| \|  \| \| \| 108 \| የባለቤትዎየትምህርት ደረጃ \| 1. መደበኛ ትምህርት ያልተማሩ  2. አንደኛ ደረጃ(1-8)  3. ሁለተኛ ደረጃ(9-12)  4. ኮሌጅ እና ከዚያ በላይ \|  \| \| \| \| \| 109 \| የባለቤተዎ የስራ ሁኔታ ምንድን ነዉ \| 1. አርሶ አደር 2. የግል ተቀጣሪ 3. የመንግስት ተቀጣሪ 4. ነጋዴ 5. ተማሪ 6. የቀን ሰራተኛ 7. ሌላ ካለ ይግለፁ-- \|  \| \| \| \| | | | | | | |  |
|  | | | | | |  | |
| 200 | አማካኝ የወር ገቢ | | ----ኢ.ብ | |  |  |  |
| **ክፍል2፡-የጤና አገልግሎት አሰጣጥን በተመለከተ** | | | | | |  |  |
| 201  202 | ያንችን ጤና እንክብካቤ ለማግኘት በዋነኛነት የመወሰን አድሉ ያለዉ ከቤተሰብ ዉስጥ ማን ነዉ?  ካንድ በላይ መምረጥ ይቻላል  ብዙ ጊዜ የሚጠቀሙበት ጤና ጣቢያ በግምት ከቤትዎ ምን ያህል ሰኣት ይጨርሳል? | | 1. እኔ 2. ባለቤቴ 3. ሌላ ሰዉ   …..ደቂቃ | |  |  |  |
| 203 | ወሊድን በተመለከተ ድንገተኛ ህመም ቢከሰት አምቡላንስ በተሎ የማግኘት እድሉ አለሽ? | | 1. አዎ 2. የለኝም | |  |  | |
| **ክፍል3፡-፡ከወሊድ ጋር የተያያዙ ጥያቄዎች**   \| 301 \| ስንተኛ እርግዝናዎት ነበረ ያሁኑ ልጅዎት ? \| ---- \|  \| \| --- \| --- \| --- \| --- \| \| 302 \| ልጅ ጠፍቶ ብዎት ያዉቃል ካሁኑ ልጅዎ በፊት? \| 1. አዎ 2. የለም \| የለም ከሆነ ወደ ጥያቄ ቁትር \| \| 303 \| በየትኛዉ ችግር ነበር ልጅዎ የጠፋዉ?  ካንድ መመለስ ይቻላል \| 1. በእርግዝና ወቅት ከ28 ሳምንት በፊት የጠፋ 2. ከሆድ ዉስጥ ሞቶ የወጣ 3. ተወልዶ ከ2ሰአት ቆይታ በፊት 4. ተወልዶ ከ2 ሰአት እስከ   7 ቀን ባለዉ \|  \| \| 304 \| እርግዝናን ተያይዘዉ በሚመጡ በሽታወች ታመዉ ነበር ያሁኑን ልጅዎትን እንዳረገዙ? \| 1. አዎ 2. የለም \| የለም ከሆነ ወደ ጥያቄ \| \| 305 \| በየትኛዉ በሽታ ነበር?  ካንድ በላይ መልስ ይቻላል \| 1. በማህፀን ደም መፍሰስ 2. የደም ግፊት መጨመር 3. ሌላ ካለ … \|  \| \|  \|  \|  \|  \| \|  \|  \|  \|  \| \|  \|  \|  \|  \| \|  \|  \|  \|  \| \|  \|  \|  \|  \| \|  \|  \|  \|  \|   **ክፍል4፡-ስለ እናቶች የጤና እንክብካቤ አገልግሎትን በተመለከተ**   \| 401 \| ስንተኛዉ ሳምንት ላይ ነበር ማርግዝሽን ያረጋገጥሽ? \| -----ሳምንት  ------ኣላዉቀዉም \|  \| \| --- \| --- \| --- \| --- \| \| 402 \| ስንተኛዉ ሳምንት ላይ የመጀመሪያ ክትትል ጀመሩ? \| ----ሳምንተ  -----ኣላዉቀዉም \|  \| \| 403 \| የቅድመ ወሊድ ክትትል የትነዉ ያደረጉት? \| 1. ጤና ኬላ 2. ጤና ጣቢያ 3. ሆሰፒታል 4. ከግል ክሊኒክ \|  \| \| 404 \| ስንት ጊዜ የቅድመ ወሊድ ክትትል አደረግሽ? \| ----ጊዜ \|  \| \|  \|  \|  \|  \| \|  \|  \|  \|  \| \|  \|  \|  \| \|  \|  \|  \|  \| \|  \|  \|  \|  \| \|  \|  \|  \|  \| \|  \|  \|  \|  \| \|  \|  \|  \|  \| \|  \|  \|  \|  \| \|  \|  \|  \|  \| \| 405 \| የት ነበር የመጨረሻዉን ልጅ የወለዱት? \| 1. ጤና ተቋም  2. ቤት  3. ሌላ ካለ ይጠቀስ \| ከጢና ተቋምዉ  ጭ ከወለዱ ወደ  ጥያቄ ቁ 407 \| \| 406 \| ከጤና ተቋም ከወለዱ ከየት \| 1. ጤና ኬላ 2. ጤና ጣቢያ 3. የመንግስት ሆስፒታል 4. የግል ክሊኒክ \|  \| \| 407 \| ሲወልዱ ማን ነበር ያዋለደዎት? \| 1. ሀኪም 2. ነርሰ 3. ሚድዋየፍ 4. ጤና ኤክስ ቴንሽን \|  \| \| 408 \| ከወለዱ በኃላ የድህረ ወሊድ ክትትል አገልግሎት አግኝተዋል? ከ ጤና ተቃሙ ሳይወጡ ወይም ከወጡ በሁአላ \| 1. አዎ  2. የለም \|  \| \| 409 \| አዎ ካሉ መቼ መቼ ነበር የድህረ ወሊድ ክትትል አገልግሎት ያገኙት?  ከአንድ በላይ ምርጫ ይቻላል \| 1. በ 24 ሰአት ዉስጥ  2. ከ 2-3 ቀን ባለዉ ጊዜ  3. ከ 6-7 ቀን ባለዉ ጊዜ  4. በ 6ኛዉ ሳምንት \|  \| \| 500 \| ሲወልዱ ማን ነበር ያዋለደዎት? 1. \| 1. ሀኪም  2. ነርሰ  3. ሚድዋየፍ  4. ጤና ኤክስ ቴንሽን  5. የልምድ አዋላጆች \|  \| \| 501 \| ከወለዱ በኃላ የድህረ ወሊድ ክትትል አገልግሎት አግኝተዋል? \| 1. አዎ  2. አላገኘሁም \|  \| \| 502 \| አዎ ካሉ መቼ መቼ ነበር የድህረ ወሊድ ክትትል አገልግሎት ያገኙት?  ከአንድ በላይ ምርጫ ይቻላል \| 1. በ 24 ሰአት ዉስጥ  2. ከ 2-3 ቀን ባለዉ ጊዜ  3. ከ 6-7 ቀን ባለዉ ጊዜ  4. በ 6ኛዉ ሳምንት \|  \| \| 503 \| ምን አይነት ጤና ተቋም ነበር የድህረ ወሊድ ክትትል አገልግሎት የሚያገኙት? \| ከአንድ በላይ ምርጫ ይቻላል   1. ጤና ኬላ 2. ጤና ጣቢያ 3. ከመንግስት ሆስፒታል 4. ከግል ጤና ተቋም \|  \|   አመሰግናለሁ | | | | | | |  |
